# Predicting the recurrence and overall survival of patients with glioma based on histopathological images using deep learning

**DOI:** 10.3389/fneur.2023.1100933

**Published:** 2023-03-31

**Authors:** Chenhua Luo, Jiyan Yang, Zhengzheng Liu, Di Jing

**Affiliations:** ^1^Department of Oncology, National Clinical Research Center for Geriatric Disorders, Xiangya Hospital, Central South University, Changsha, China; ^2^Xiangya School of Medicine, Central South University, Changsha, China

**Keywords:** glioma, pathomics, deep learning, recurrence, overall survival

## Abstract

**Background:**

A deep learning (DL) model based on representative biopsy tissues can predict the recurrence and overall survival of patients with glioma, leading to optimized personalized medicine. This research aimed to develop a DL model based on hematoxylin-eosin (HE) stained pathological images and verify its diagnostic accuracy.

**Methods:**

Our study retrospectively collected 162 patients with glioma and randomly divided them into a training set (*n* = 113) and a validation set (*n* = 49) to build a DL model. The HE-stained slide was segmented into a size of 180 × 180 pixels without overlapping. The patch-level features were extracted by the pre-trained ResNet50 to predict the recurrence and overall survival. Additionally, a *light-strategy* was introduced where low-size digital biopsy images with clinical information were inputted into the DL model to ensure minimum memory occupation.

**Results:**

Our study extracted 512 histopathological features from the HE-stained slides of each glioma patient. We identified 36 and 18 features as significantly related to disease-free survival (DFS) and overall survival (OS), respectively, (*P* < 0.05) using the univariate Cox proportional-hazards model. Pathomics signature showed a C-index of 0.630 and 0.652 for DFS and OS prediction, respectively. The time-dependent receiver operating characteristic (ROC) curves, along with nomograms, were used to assess the diagnostic accuracy at a fixed time point. In the validation set (*n* = 49), the area under the curve (AUC) in the 1- and 2-year DFS was 0.955 and 0.904, respectively, and the 2-, 3-, and 5-year OS were 0.969, 0.955, and 0.960, respectively. We stratified the patients into low- and high-risk groups using the median hazard score (0.083 for DFS and−0.177 for OS) and showed significant differences between these groups (*P* < 0.001).

**Conclusion:**

Our results demonstrated that the DL model based on the HE-stained slides showed the predictability of recurrence and survival in patients with glioma. The results can be used to assist oncologists in selecting the optimal treatment strategy in clinical practice.

## 1. Introduction

Glioma is the most common central nervous system (CNS) tumor, accounting for 30% of primary CNS tumors (1). According to the 2021 WHO classification of CNS tumors, gliomas can be classified into multiple subtypes based on histological features and the expression of specific molecular proteins (2). Gliomas derived from precursor or glial cells are divided into WHO I–IV grades (3, 4), and the tumor grades and molecular biology examination are closely related to recurrence and prognosis. The precise prediction of DFS (recurrence) and OS (survival) can provide solid instruction for treatment design and clinical practice. The accurate prediction of OS and DFS remains a challenge for both pathologists and oncologists because of tumor heterogeneity. It is necessary to develop an efficient and labor-saving approach to predict the recurrence and prognosis of gliomas, thus optimizing treatment plans (5, 6).

Histopathology is the gold standard in diagnosing CNS tumors and provides guidelines for the selection of treatment options. Currently, pathologists face a series of complicated diagnostic criteria, which mainly depend on subjective judgment and the experience of senior pathologists.. van den Bent et al. (7) demonstrated that interobserver variation has led to the differential diagnosis from the same histopathological slide and led to an inaccurate prognosis. With the development of digital pathology, whole slides imaging (WSI) provides an approach to acquiring high-resolution images to assist oncologists in detecting atypical micro-lesions (8). In recent years, convolutional neural networks (CNNs) have been utilized to analyze image characteristics in fields such as grading glioma from histopathological slides (9, 10), predicting the recurrence *via* intraoperative simulated Raman histology (11), and detecting the microvasculature (12), which boosts the development of quantitative image assessment in glioma (13). So far as we know, there is little research related to predicting the recurrence and survival in patients with glioma by pathological WSI using the deep learning method.

**Figure 1:**
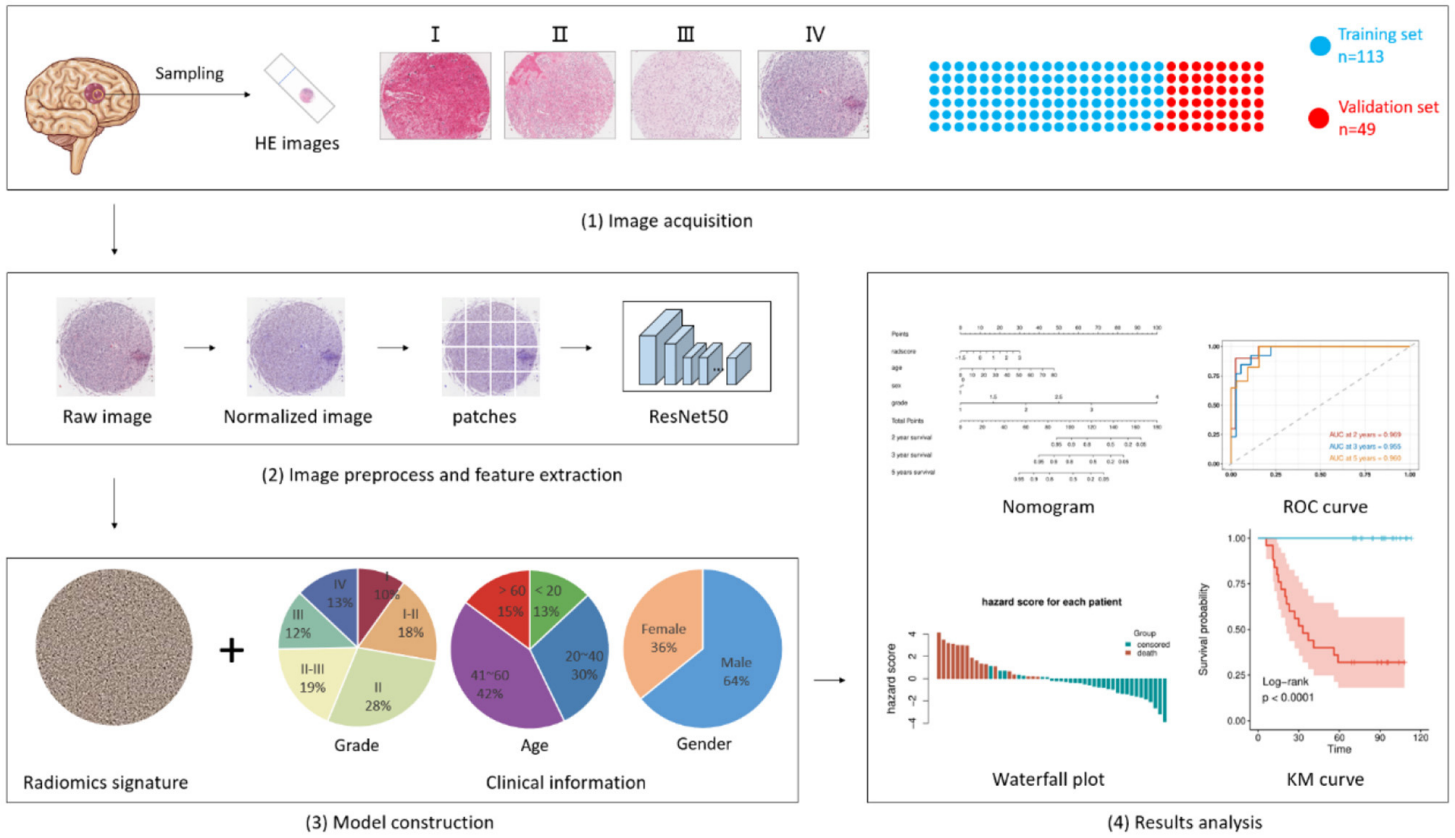
Flowchart of pathomics and clinical-pathomics model construction. i. Biopsy samples acquired from 162 patients were scanned and uploaded to form a training set (*n* = 113) and a validation set (*n* = 49). ii. Digital images were segmented into patches and inputted into pre-trained ResNet50 for feature extraction. An integrative pathomics signature was generated in this process. iii. The pathomics signature along with several clinical characteristics was fed into another model for model construction. iv. results were collected and analyzed. The Kaplan-Meier curve was used to estimate DFS and OS. The time-dependent receiver operating characteristic (ROC) curves, along with nomograms, were used to assess the diagnostic performance.

In our research, a CNN was used to analyze the representative HE-stained biopsy tissues and combined with clinical information, including age, sex, and grading, to predict recurrence and OS in patients with glioma (Figure 1). The HE-stained slides contain the potential information that indicates the differentiation and malignancy of tumors (10, 14–16). The WHO classification also contains a lot of information that might give clues to predicting the clinical outcome of patients with glioma, so it is critically weighted in the analysis of clinical data. The innovation in our study focused on the keyword *light*. Conventional WSIs of glioma HE-stained slide take approximately 10 min each, taking up more than 2GB storage space, whereas scanning the representative biopsy tissue only needs 1 min per slide and occupied approximately 200 MB of storage space. The powerful predictive capability and relatively smaller files are easy for consultation and transfer between medical centers and artificial intelligence centers. Therefore, we built a predictive deep learning (DL) model using pathomics signatures in combination with clinical information to predict the recurrence and OS of glioma.

## 2. Materials and methods

### 2.1. Study population

The retrospective study included 162 post-operation patients with glioma from January 2010 to January 2021 and was approved by the Medical Ethics Committee of Xiangya Hospital. Patients were selected according to the following criteria: (i) patients were diagnosed with glioma by pathological examination, (ii) patients were treated with surgery, and (iii) patients were followed up every 3 months after surgery, and recorded detailed information was obtained. The exclusion criteria were as follows: (i) patients diagnosed with other malignant tumors, and (ii) pathological images with inferior quality or incomplete clinical information, including sex, age, and grade. A total of 162 patients with glioma were randomly divided into a training set (*n* = 113, 70%) and a validation set (*n* = 49, 30%).

### 2.2. Image acquisition and preprocess

For the histopathological image of each patient, we first applied normalization to reduce the variances introduced during image acquisition, tissue processing, staining, etc. In this step, a standard histopathological image was chosen as a reference, and a GPU-accelerate stain normalization tool, torchstain (https://github.com/EIDOSLAB/torchstain), was used to accomplish this. Then, the histopathological image was converted from RGB to gray, and the tumoral area was segmented based on the threshold generated by Otsu's method. A *light-strategy* was introduced where low-size digital biopsy images with clinical information were inputted into the DL model to ensure minimum memory occupation. Patches with a size of 180 × 180 without overlap were extracted from each histopathological image. The patches with <80% tumoral area were deemed as background and were excluded. After those two steps, a total of 8,971 patches were generated and thereafter used in our following analysis.

### 2.3. Feature extraction, model development, and model validation

A pre-trained ResNet 50 was used to extract patch-level features. The features of the last convolutional layer were extracted and fed into a global average pooling layer. Then, we averaged the patch-level features over the patches for each patient and used the pooling features for subsequent analysis.

Before model development, a univariate Cox proportional-hazards model was used to test the significance of each feature, only the features with a *p* < 0.05 were retained. These were then fed into a multivariate proportional-hazards model to generate a pathomics signature. Then, the pathomics signature, along with several clinical characteristics (age, sex, and grade), were fed into another multivariate proportional-hazards model for clinicopathologic model development. In addition, to investigate the incremental value of the pathomics signature, we constructed a model using merely clinical characteristics.

### 2.4. Statistical analysis

Statistical analysis and model development were conducted using R (version 4.0.3) and Python (version 3.7.10). The Kaplan-Meier curve and log-rank test were used to estimate DFS and OS. The C-index was used to measure the concordance between the model prediction and the observed outcomes. It ranges from 0.5, which indicates a random prediction, to 1.0, which indicates a perfect prediction. A higher C-index value indicates that the model is better at predicting patient outcomes. In addition, the time-dependent receiver operating characteristic (ROC) curves, along with nomograms, were used to assess the diagnostic performance at a fixed time point (1/2 years DFS, 2/3/5 years OS). A two-sided *P* < 0.05 was considered significant.

## 3. Results

### 3.1. Clinicopathologic characteristics

The clinicopathologic characteristics for the training and validation set are listed in Table 1. Of the 162 patients included in our study, 104 (64.20%) were men, and the median age of all patients was 41 (2–80) years. In the training set, the median survival time for DFS and OS were 62 (9–113) and 84 (9–13) months, respectively. In the validation set, the median survival time for DFS and OS were 63 (6–113) and 76 (6–13) months, respectively. There was no conspicuous difference between training and validation sets in terms of clinicopathological features or survival time.

**Table 1:** The clinicopathological features of 162 patients with glioma.

**Parameters**	**Training set (*n* = 113)**	**Validation set (*n* = 49)**	***p*-value**
**Sex, No [%]**			0.05
Men	67 [59.29]	37 [75.51]	
Women	46 [40.71]	12 [24. 49]	
**Age, No [%]**			0.80
≤20	14 [12.39]	7 [14.29]	
21–40	32 [28.32]	16 [32.65]	
41–60	49 [43.36]	20 [40.82]	
≥61	18 [15.93]	6 [12.24]	
**Grade, No [%]**			0.70
I	13 [11.50]	3 [6.12]	
II	49 [43.36]	26 [53.06]	
III	37 [32.74]	13 [26.53]	
IV	14 [12.39]	7 [14.29]	
**Subtype, No [%]**			0.14
Astrocytoma	35 [29.41]	8 [16.33]	
Oligodendroglioma	4 [3.36]	2 [4.08]	
Ependymoma	1 [0.84]	2 [4.08]	
Ganglioglioma	5 [4.20]	2 [4.08]	
Glioblastoma	74 [62.18]	35 [71.43]	
**DFS (months)**			0.53
Median (range)	62 (9–113)	63 (6–113)	
**OS (months)**			0.38
Median (range)	84 (9–113)	76 (6–113)	

### 3.2. Model development and validation

A total of 512 histopathological features were extracted from each patient, of which, 36 and 18 features were identified as significantly related to DFS and OS (*P* < 0.05) using the univariate Cox proportional-hazards model. As shown in Table 2, in the validation which consists of 49 patients, the pathomics signature constructed by those features showed a C-index of 0.630 and 0.652 for DFS and OS prediction, respectively. Meanwhile, the clinical model showed a C-index of 0.839 and 0.897, respectively. If the pathomics signature was combined with clinical characteristics, the clinicopathologic model showed an improved C-index of 0.859 and 0.928 for DFS and OS prediction, respectively.

**Table 2:** The C-index of the different models on the validation set.

	**DFS**	**OS**
Pathomics signature	0.630	0.652
Clinical model	0.839	0.897
Clinicopathologic model	0.859	0.928

**Figure 2:**
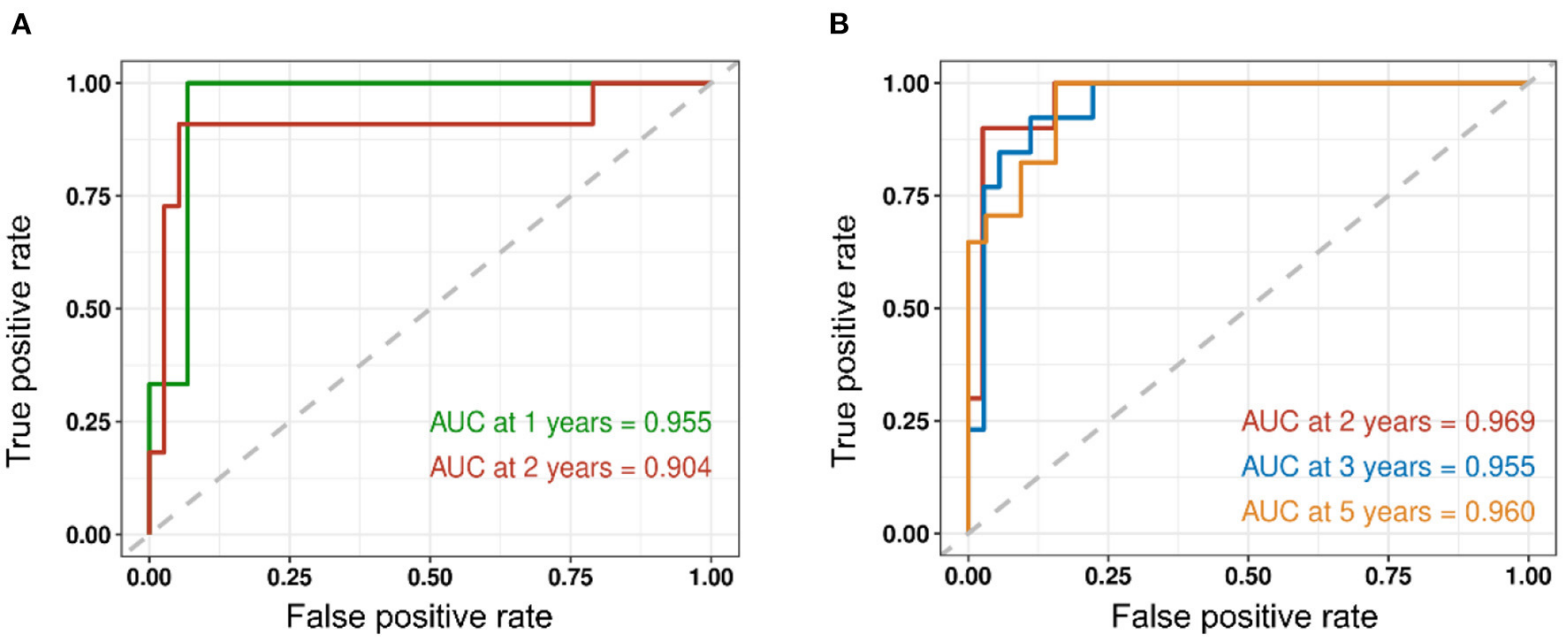
Time-dependent receiver operator characteristics (ROC) analysis of recurrence and survival is shown. **(A)** The 1- and 2-year DFS were 0.955 and 0.904, respectively. **(B)** The 2-, 3-, and 5-year OS were 0.969, 0.955, and 0.960, respectively.

**Figure 3:**
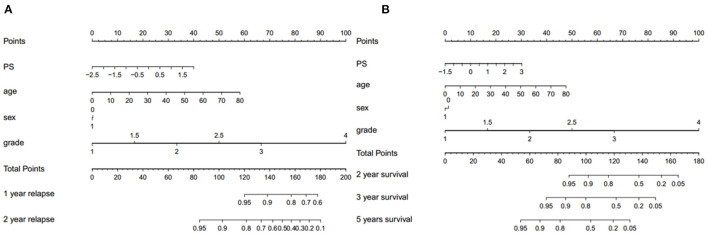
The corresponding nomogram showing the contribution of different factors. “PS” refers to the pathomics signature, “Points” refers to the numerical value assigned to each predictor variable based on its current value, and “Total points” refers to the sum of the points for all predictor variables. **(A)** Recurrence and **(B)** survival.

We assessed the predictive performance of the clinicopathologic model using time-dependent receiver operator characteristics (ROC) analysis at different follow-up times (Figure 2). The 1- and 2-year DFS were 0.955 and 0.904, respectively; the 2-, 3-, and 5-year OS were 0.969, 0.955, and 0.960, respectively. The corresponding nomogram is shown in Figure 3; “PS” refers to the pathomics signature, “Points” refers to the numerical value assigned to each predictor variable based on its current value, and “Total points” is the sum of the points for all predictor variables, which is used to calculate the overall predicted outcome.

**Figure 4:**
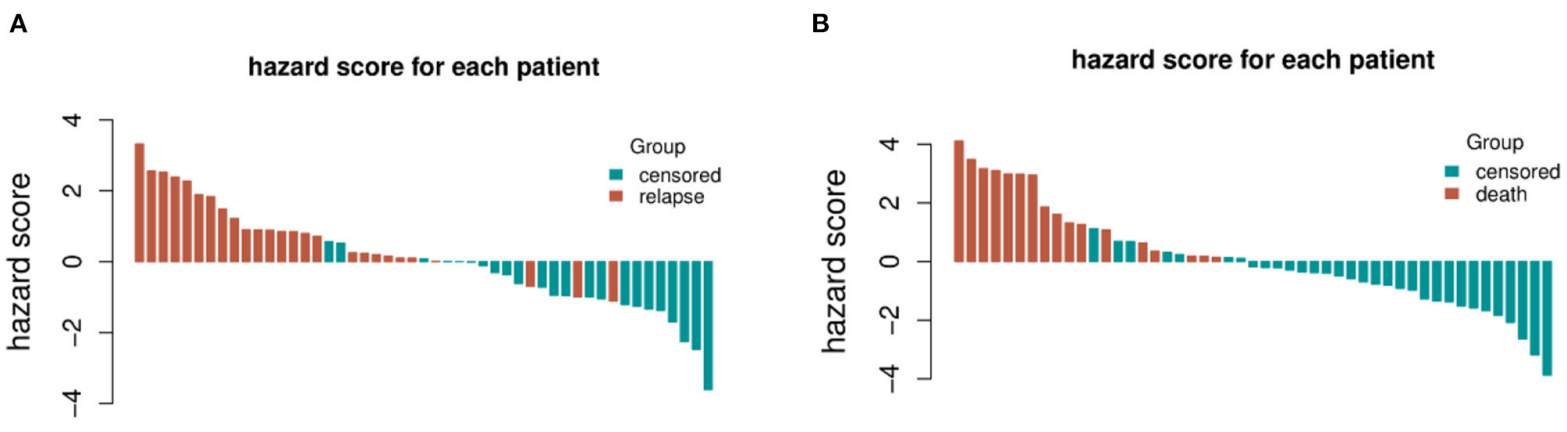
The distribution of hazard score and survival status for each patient included in the validation set. **(A)** Recurrence and **(B)** survival.

**Figure 5:**
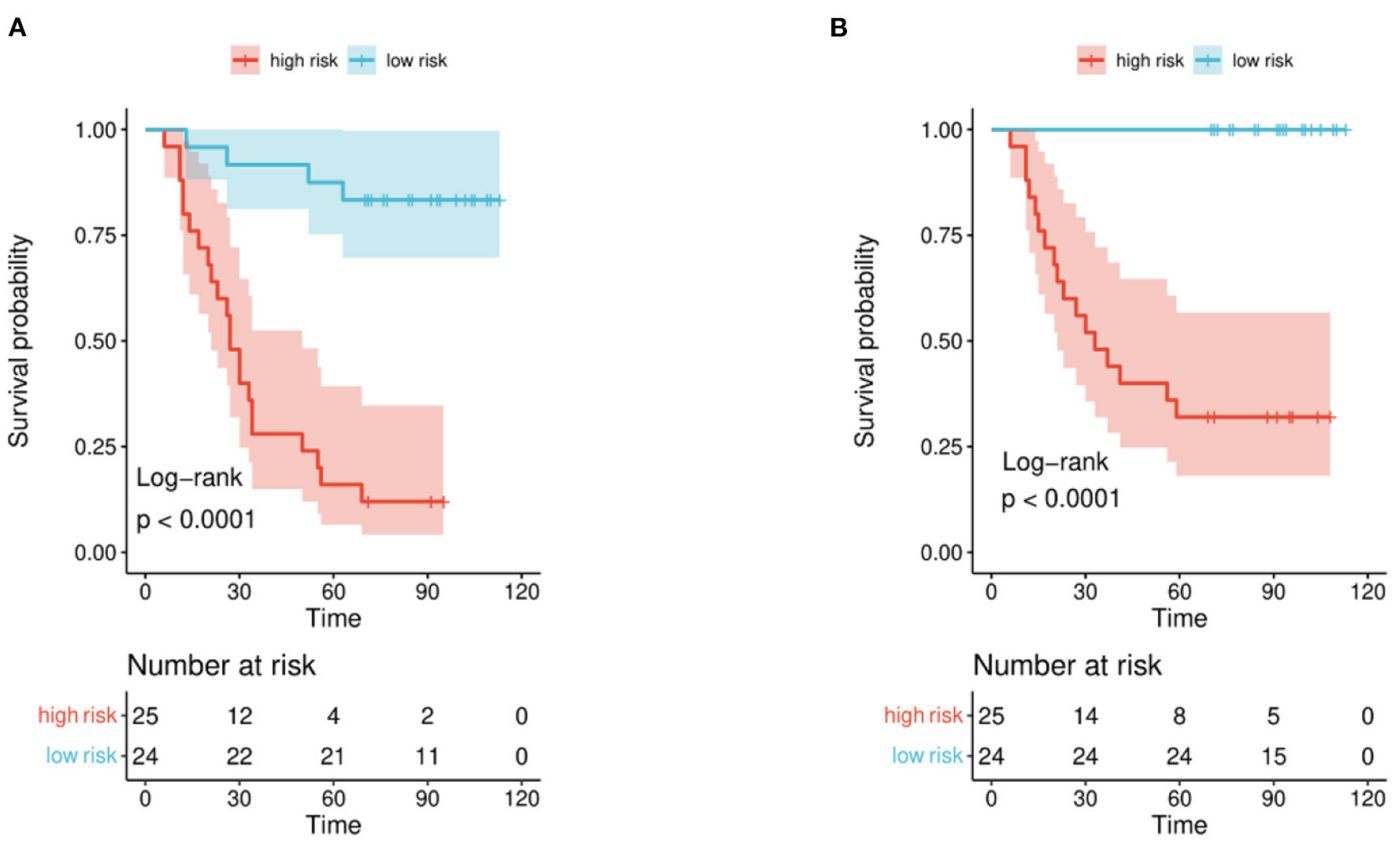
Kaplan-Meier curve for patients in the validation set. Patients in the set were stratified into high-risk and low-risk groups. **(A)** Recurrence and **(B)** survival.

The distribution of hazard score and survival status is shown in Figure 4. Patients with higher scores generally had worse survival status than those with lower scores. We stratified the patients into low- and high-risk groups (Figure 5) using the median hazard score (0.083 for DFS and −0.177 for OS). The Kaplan-Meier curve showed that a significant difference existed between these groups (*P* < 0.001).

**Figure 6:**
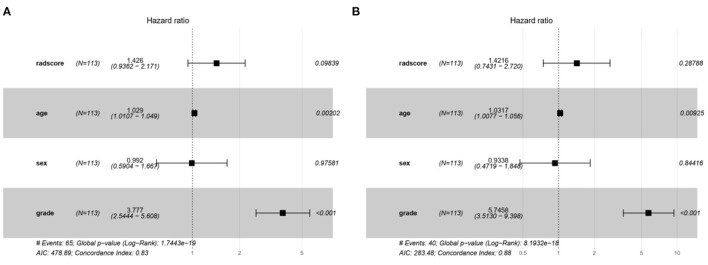
The forest plots of characteristic risk scores to analyze the recurrence and survival outcome in the DL model. **(A)** Recurrence and **(B)** survival.

The characteristic risk scores to analyze the recurrence and survival outcome in the DL model are shown in the forest plots (Figure 6).

## 4. Discussion

In this research, we used a combination of pathomics features and clinical information to build a DL model for the prediction of glioma recurrence and OS. The DL model provided an efficient approach to outcome prediction, achieving a C-index of 0.859 in the recurrence prediction model and 0.928 in the OS prediction model. The WHO classification is currently the authority international standard in the pathological diagnosis of glioma, which can predict the outcome of patients. The WHO classified CNS tumors into four grades (I-IV) (3, 4), the higher the grade, the poorer prognosis of the tumor. The WHO classification for CNS tumors was recently updated to its fifth edition in 2021 (2), which advanced the role of molecular diagnosis and emphasized the significance of integrated diagnoses and layered reports. Therefore, the WHO classification was of pivotal weight in the analysis of clinical information to sufficiently detect the hidden information in the tumor grade.

Several studies have used various methodologies to develop models to predict the clinical outcome of patients with glioma. Choi et al. (17) proved in their research that radiomics analysis achieved an AUC of 0.709 to predict survival in lower-grade gliomas. Mobadersany et al. (18) illustrated an approach with their survival convolutional neural networks(SCNN), which could integrate data from WSIs and genomic biomarkers, to predict the OS of patients with glioma. Yan et al. (19) developed a diffusion tensor imaging (DTI)-based deep learning signature to predict OS in patients with infiltrative gliomas. However, to date, little research has been conducted that uses deep learning methods in pathological WSIs to predict at the same time both survival and recurrence.

Pathological methods as the gold standard have a wide application in differentiating pseudoprogression, necrosis, and recurrence (20). Tissue sampling that contains much-hidden information is becoming progressively crucial in the era of personalized medicine (11, 21). With the rising number of patients suspected of glioma, the complicated labor-consuming workflow where every slide image is manually examined is inefficient in clinical practice (22–25). Moreover, the uneven distribution and labor shortages of experienced neuropathologists have been a barrier to examining every intracranial biopsy in glioma (26, 27). Advances in CNNs and high-resolution WSIs are providing more diagnosis opportunities by improving the work efficiency for neuropathologists *via* AI-assistant. In this study, the HE-stained slides of representative biopsy tissues were analyzed by state-of-the-art CNNs to increase the accuracy of recurrence detection and reveal the survival information in gliomas.

Analyzing original WSIs data from multiple medical centers has been an obstacle because of limited storage volume and transmission speed, with some publicly available datasets such as TCGA containing large WSIs, each exceeding 2 GB in size. Jin et al. (10) improved this situation by developing a specially designed slide scanner that can automatically divide each WSIs into 300 patches. Cheng et al. (28) invited three subspecialists to manually stretch out regions of interest (ROIs) in images, which is convenient but labor-intensive. Our study focused on the *light-strategy* that could acquire images from the representative biopsy tissues in a short period. This strategy brings the following advantages: (i) reducing the imaging time to <1 min, (ii) the small-size biopsy limited the total storage to under 1 GB, and (iii) the compressed files were convenient for data transmissions, which boost multi-center cooperation in our further study.

The result of our DL model demonstrated relatively high precision for its prediction tasks but still has potential for improvement: (i) the random deviation of specimen processing of the HE-stained slide might affect the prediction accuracy in the analyzing model, (ii) the heterogeneity of glioma brought difficulties in the analysis, thus a larger dataset is required to improve performance and avoid occasionality, and (iii) multi-center datasets need to be included to further validate the performance of our DL model. Here we propose two points for improvement. First, we can consider using the DeepSurv model as an alternative to the traditional Cox model. DeepSurv is a deep learning model specifically designed for survival analysis and can better capture non-linear relationships between features and survival times. Secondly, instead of pre-training the feature extraction model on ImageNet, we can consider pre-training it on pathological images to better capture the specific features relevant to our investigation. These modifications could potentially lead to improved performance and more accurate predictions in our survival analysis.

In conclusion, DL models based on clinicopathological characteristics and pathomics signature had a superior predictive ability for recurrence and overall survival in patients with glioma and showed greater value for personalized treatment.

## Data availability statement

The raw data supporting the conclusions of this article will be made available by the authors, without undue reservation.

## Ethics statement

The studies involving human participants were reviewed and approved by the Ethics Committee of Xiangya Hospital. The patients/participants provided their written informed consent to participate in this study. Written informed consent was obtained from the individual(s) for the publication of any potentially identifiable images or data included in this article.

## Author contributions

DJ and CL designed the project. CL and JY collected the clinical data. CL analyzed the data and drafted the original manuscript. ZL as the senior professor evaluated the patients with glioma. DJ revised this manuscript. All authors reviewed this manuscript and approved this submission.
