# Corrigendum: Predicting the recurrence and overall survival of patients with glioma based on histopathological images using deep learning

**DOI:** 10.3389/fneur.2023.1209701

**Published:** 2023-05-10

**Authors:** Chenhua Luo, Jiyan Yang, Zhengzheng Liu, Di Jing

**Affiliations:** ^1^Department of Oncology, National Clinical Research Center for Geriatric Disorders, Xiangya Hospital, Central South University, Changsha, China; ^2^Xiangya School of Medicine, Central South University, Changsha, China

**Keywords:** glioma, pathomics, deep learning, recurrence, overall survival

In the published article, there was an error in affiliations for Jiyan Yang, Zhengzheng Liu and Di Jing. Instead of “Chenhua Luo^1, 2^, Jiyan Yang^1^, Zhengzheng Liu^2^ and Di Jing^2*^”, it should be “Chenhua Luo^1, 2^, Jiyan Yang^2^, Zhengzheng Liu^1^ and Di Jing^1*^”.

The authors apologize for this error and state that this does not change the scientific conclusions of the article in any way. The original article has been updated.

